# Genetically modified pigs with α1,3-galactosyltransferase knockout and beyond: a comprehensive review of xenotransplantation strategies

**DOI:** 10.3389/fimmu.2025.1663246

**Published:** 2025-11-04

**Authors:** Ewelina Stelcer, Anna Wozniak, Dorota Magner, Joanna Zeyland

**Affiliations:** Department of Biochemistry and Biotechnology, Poznan University of Life Sciences, Poznan, Poland

**Keywords:** alpha-1,3-galactosyltransferase, alpa-Gal epitope, xenotransplantation, hyperacute rejection (HAR), anti-Gal specific antibodies

## Abstract

Xenotransplantation holds promise to eliminate the shortage of organs intended for humans in need. Pigs constitute the most suitable organ xenograft donor due to the fact that their organ anatomy physiological metabolism and immune system resemble those of humans. However, swine organs rapidly cause hyperacute rejection (HAR) and acute humoral xenograft rejection (AHXR) after transplantation. HAR and AHXR are caused by the presence of xenoreactive natural immunoglobulins directed toward a galactose alpha1-3-galactose (alpha-Gal) epitope on porcine vascular endothelium. In order to suppress both types of rejection, pigs with alpha1,3-galactosyltransferase gene knockout (GT-KO) and other genetic modifications (like simultaneous expression of the human complementary regulatory proteins) are intensively investigated. This review highlights the usefulness of GT-KO pig – derived organs such as kidney, heart, corneal, and lung in xenotransplantation. To obtain transgenic pigs researchers can use several techniques based on pronuclear and cytoplasmic microinjection, somatic cell nuclear transfer (SCNT), viral transduction of DNA and DNA transposable element -based technology, site specific nucleases and modifications of the CRISPR/Cas bacterial immune system. Some additional strategies like targeted immunosuppression or tolerance induction of B and T cells will be essential for sustained survival of xenografts. Although xenotransplantation with the use of pigs is a very rapidly evolving field, more research is needed to create perfectly compatible with the human immune system organs.

## 1 Introduction

Animal organs, tissues and cells constitute a promising strategy in xenotransplantation. In particular, pig organs are studied in detail because of their numerous features: (i) they are similar to humans in anatomical size and structure, immunology, genome, and physiology, (ii) they can be bred on demand, (iii) and the donor pig must be free of specific pathogens that might potentially lead to morbidity in the recipient (1). Vascularized organ xenografts are highly susceptible to hyperacute rejection (HAR). It is caused by antibodies produced by the human body which bind to the vascular endothelium of the xenograft. This phenomenon leads to the activation of the complement and coagulation systems. In the pig-to-human configuration, the key target for human xenoreactive antibodies is the carbohydrate disaccharide antigen galactose-alpha-1,3-galactose (Galα1–3Gal, alpha-Gal) which is present in porcine tissues (2). Consequently, for successful xenotransplantation, novel drugs must be invented to arrest the production by B lymphocytes of anti-alpha-galactose antibodies, clear the existing antibodies and thus inhibit organ damage (3).

**Figure 1:**
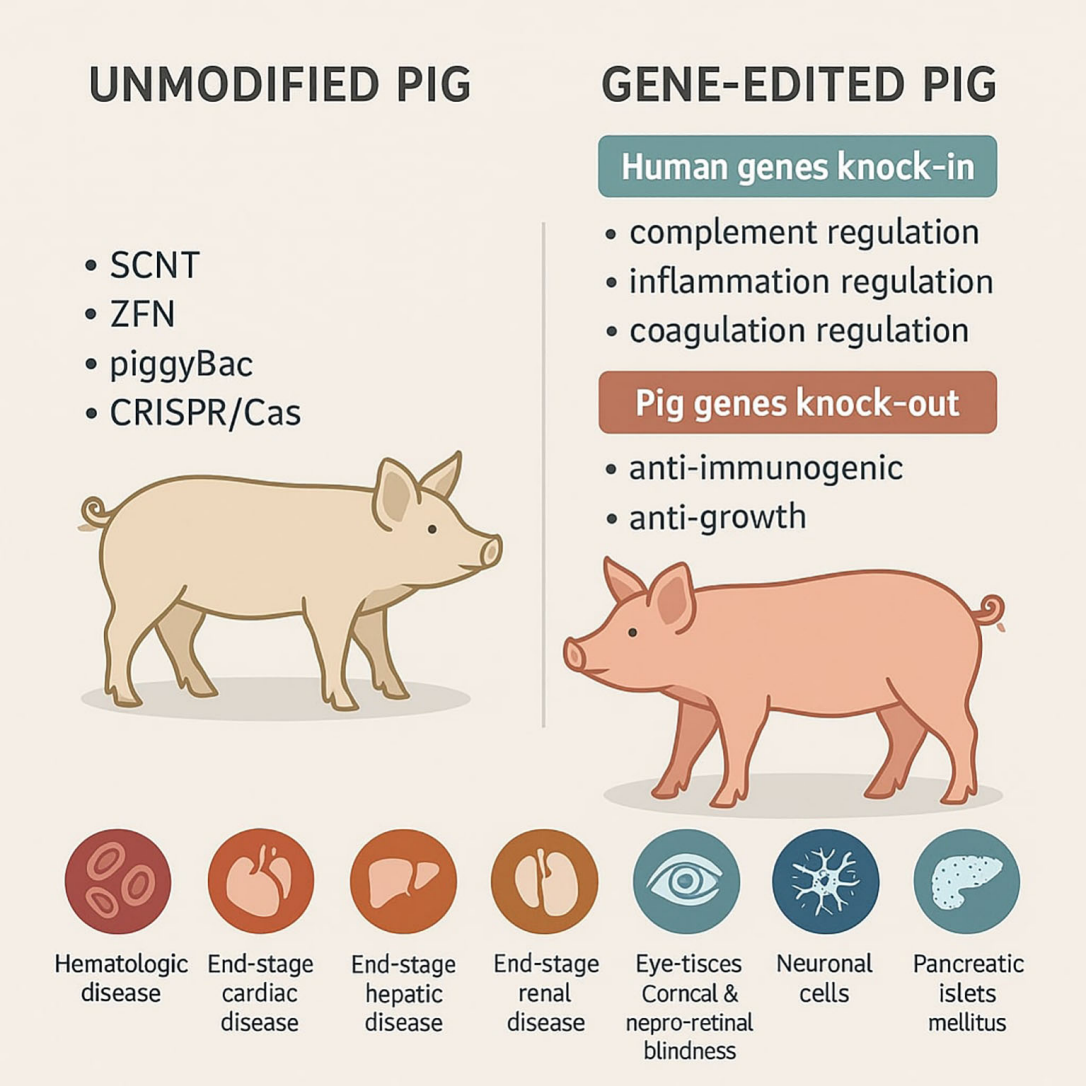
Graphical summary of genetic engineering methods used to obtain transgenic pigs for biomedical purposes.

Galα1–3Gal is an oligosaccharide which is responsible for glycosylation of various proteins of non-primate mammals. In humans, apes and old-world monkeys alpha-1,3- galactosyltransferase is inactive and thus these species do not express alpha-Gal epitope (the primate species lost expression of alpha-Gal epitope several million years ago because of genetic mutation). The lack of alpha-Gal led to the production of antibodies against this - from that moment - “foreign” antigen in primates. These and other “natural” antibodies “are produced in response to alpha-Gal-expressing pathogens that colonize the primate’s gastrointestinal tract during neonatal life (4). Hence, primates produce IgM and IgG antibodies (which constitute approximately 1% of circulating immunoglobulins) targeted to this oligosaccharide and consequently leading to graft rejection of pig organs. In xenotransplantation involving pig organs, these antibodies constitute a pivotal immune barrier (**Figure 1**) (5, 6).

The well-established protocols resulting in creation of viable pigs with homozygous deletion of alpha-1,3-Gal transferase gave hope for overcoming both HAR and acute humoral xenograft rejection (AHXR). However, it is still important to bear in mind that alpha-Gal-deficient porcine xenografts can be still strongly rejected by T cells and the use of nonspecific immunosuppressive drugs in turn, may lead to severe toxicity (7). There is evidence that once the alpha-Gal epitope is abolished in (GGTA1 gene knockout, GT-KO) pigs, they synthesize anti-Gal at titers even higher than in humans. This is another indication that exclusion of the GGTA1 gene in ancestral Old World primates assured the efficient production of anti-Gal, possibly as a protective antibody-based mechanism against detrimental microbial agents carrying alpha-Gal epitopes (8). Another aspect of the alpha-Gal immune response involves anti-alpha-Gal antibodies of the IgE isotype, which have been shown to carry a high risk of anaphylaxis (9). Alpha-Gal moieties are widespread and thus easily detectable in non-primate mammals (e.g. cows, pigs, and sheep) as well as they can also be found in many food products originated from those animals (dairy products, meat, and their innards). Consumption of those products is associated with the risk of occurrence of numerous symptoms ranging from urticaria to life-threating anaphylaxis (10).

There is a strategy which involves additional expression of human H transferase which leads to the diminished binding of anti-alpha-Gal specific antibodies, thus avoiding the progress of HAR. This approach also leads to the inhibited response of natural killer (NK) cells and monocytes. The use of alpha-galactosidase (GLA), that catalyzes the removal of the alpha-D-galactose unit from the alpha-Gal antigen also may lead to the decrease in the surface presence of the antigen on transplant donor cells (11). Not only alpha-1,3 galactosyltransferase is able to synthesize alpha-Gal. One of the main candidates for the production of this epitope is iGb3 synthase (iGb3S) that belongs to the ABO blood group glycosyltransferase family. Consequently, this enzyme may have an impact on the survival of pig tissues after transplantation into humans (12). Based on literature data, the following genetic loci seem to be crucial in the transplant rejection process: epitopes (Gal, Neu5Gc, Sd(a)-like glycan), factors engaged in human complement activation (CD46,CD55, and CD59), human coagulation regulation (thrombomodulin, TBM), endothelial protein C receptor (EPCR), and vWF), endothelial protection [heme oxygenase-1 (HO-1)], and human immune regulation (macrophage inhibition- CD47, NK cell inhibition-HLA-E, and HLA-G). Those loci constitute attractive targets that after genetic modification manipulation may lead to the reduction of undesirable rejection effects (13).

Platelets constitute essential factors in survival of vascularized organ allotransplants. They have a strong impact on relations of monocytes/macrophages and T cells with endothelial cells (ECs) by production of chemokines and expression of ligands (e.g. MIP-1α, RANTES, MCP-3 and PF4). Stimulated platelets express P-selectin and CD40L (CD154). In turn, macrophages express PSGL-1 and CD40. Thus it is believed that the expression of CD154 on platelets should be highlighted in the context of xenotransplantation, since anti-CD154 monoclonal antibody is routinely investigated in immunosuppressive regimens (14).

In this context, the primary objective of the present study is to discuss available literature data regarding xenotransplantation based on pigs with GGTA1 gene knockout and primate models. This review will help to improve understanding of the process of creating genetically modified pigs with the particular emphasis on existing genetic engineering-based techniques and obtained diverse GT-KO organs. Although the topic of GT-KO pigs is not entirely new, it still constitutes the foundation of xenotransplantation.

In 2020, the U.S. FDA approved GalSafe pigs for therapeutic use including xenotransplantation. Human organ xenotransplantation remains subject to clinical research and regulatory review (15).

Thus, it is still a hot, relevant and interesting topic. Besides, this review describes other or additional genetic modifications of pigs.

Due to the fact that the use of pigs in xenotransplantation area is rapidly evolving, the authors selected and discussed the most informative in their opinion studies.

To obtain gene-edited pigs researchers have several major techniques e.g. pronuclear and cytoplasmic microinjection, somatic cell nuclear transfer (SCNT), viral transduction of DNA and DNA transposable element-based procedure (e.g. sleeping Beauty and the piggyBac systems) to choose. As a result, the obtained transgenic pigs are characterized by the presence of human genes suppressing immune response and/or the lack of pig genes responsible for graft rejection. Thanks to this, many tissues or organs like liver, kidney, heart can be theoretically successfully transplanted into human recipients in need.

## 2 Genetic modifications leading to generation of pigs deprived of alpha-Gal epitope on the cell surface

To obtain genetically modified pigs, researchers can take advantage of following techniques *inter alia* pronuclear and cytoplasmic microinjection, somatic cell nuclear transfer (SCNT), viral transduction of DNA and DNA transposable element-based method (e.g. Sleeping Beauty and the piggyBac systems). Precise genomic modifications can be achieved *via* the use of site specific nucleases: zinc finger nucleases (ZFN), transcription activator-like effector (TALE) nucleases and alterations of the CRISPR-Cas9 adapted from a naturally occurring genome editing system that bacteria use as an immune defense (16).

The site-specific nucleases induce repair of double-strand breaks (DSBs). DSBs can be repaired through action of non-homologous end joining (NHEJ) or by homologous recombination (HR) mechanisms. NHEJ often result in the creation of indel (insertion/deletion) mutations which can be used for disruption of chosen genes. On the other hand, HR uses a donor template which enables the introduction or removal of exact point mutations and the insertion of new genes (17).

Technique based on microinjection is the classic method for the creation of transgenic pigs, e.g. via pronuclear DNA microinjection to express the *GLA*, the human alpha-1,2-fucosyltransferase gene (*FUT II*), or both genetic modifications. However, this method has one major limitation which is low efficiency (about 2-3% in pigs) (18). Some of the first reports (18) concern production of transgenic male pigs, which possessed a *GGTA1* knockout allele and expressed a randomly inserted human *FUT II* transgene. For this purpose, the authors used nonisogenic DNA in preparing the *GGTA1* gene targeting constructs and designed efficient polymerase chain reaction (PCR) procedures to demonstrate targeted clones. This approach aimed to link the GGTA1 knockout genotype to a ubiquitously expressed fucosyltransferase transgene associated with the secretion of the universally tolerated H antigen (19).

Phelps et al. (19) performed SCNT with three double knockout cell lines. Firstly, they disrupted one allele of the *GGTA1* gene in cloned pigs. Then, they took advantage of a technology involving bacterial toxin selection to identify cells in which the second allele had been disrupted. Based on sequencing analysis, knockout of the second allele of the alpha-Gal gene was caused by a T-to-G single point mutation at the second base of exon 9. Those results indicate that piglets carrying a point mutation in the *GGTA1* could allow to create pigs deprived of alpha-Gal epitope free of antibiotic-resistance genes which is crucial for human safety (20).

Miniature swine with alpha-Gal knock-out (Gal ^-^/^-^ pigs) have been created by nuclear/embryo transfer from modified fibroblasts. None of the analyzed tissues revealed alpha-Gal expression. Moreover, Gal^-^/^-^ pigs produced anti-Gal antibodies that are cytotoxic to Gal ^+^/^+^ pig cells. Peripheral blood mononuclear cells (PBMCs) derived from Gal ^-^/^-^ swine did not show proliferative or cytolytic T-cell response toward Gal ^+^/^+^ swine leukocyte antigen (SLA)-matched PBMCs (21).

Kolber-Simondes et al. (21) pick spontaneous null mutant cells from fibroblast cultures of heterozygous animals necessary for the next round of nuclear transfer (NT). This serial NT has allowed to produce alpha-1,3-galactosyltransferase null piglets in a significantly shorter time than would be needed for typical breeding from heterozygotes. A frequent loss of *GGTA1* function has been reported. Healthy (hemizygous and homozygous for the gene-targeted allele), piglets, were created through NT by taking advantage of fibroblasts which previously underwent deletional and crossover/gene conversion incidents (22).

Kwon et al. (22) not only produced a transgenic piglet with GT-KO alone but also piglets simultaneously expressing multiple transgenes (based on using polycistronic vector), such as human *DAF*, *hCD39*, *hTFPI*, *hC1-INH*, and *hTNFAIP3* genes to reduce graft rejection in the primates during transplantation. However, the obtained results indicate that transgenic expression of aforementioned human genes in pigs overall attenuated hematopoiesis (but not erythropoiesis). It resulted in abnormally low numbers of platelets and white blood cells (WBCs) like neutrophils, eosinophils, basophils, and lymphocytes. On the other hand those piglets had similar numbers of red blood cells (RBC) compared to the control group (23).

Wang et al. (23) created triple gene (*GGTA1*, *CMAH*, and *b4GalNT2*) knockout (TKO) pigs using CRISPR-Cas9 targeting. In contrast to unmodified pigs, the liver, spleen, and pancreas isolated from TKO pigs were characterized by similar levels of human IgG and IgM binding, whereas TKO pig- derived corneas, heart, lung, and kidney revealed limited human IgG and IgM binding. Interestingly, they also demonstrated that the expression of Sd(a) antigen in the corneal tissue was at the higher level than that of alpha-Gal and Nue5Gc, suggesting that Sd(a) can be a key antigen detected in corneas. This phenomenon may be the reason for failed GT-KO/CD46 porcine corneal xenotransplantation into non-human primates (24).

Estrada et al. (2022) provided evidence for creation of pigs lacking *GGTA1*/*CMAH*/*β4GalNT2* genes taking advantage of CRISPR-Cas9 and gRNA technology. They assessed human, rhesus macaque, and baboon antibody binding to porcine PBMCs. PBMCs isolated from those pigs revealed diminished human IgM and IgG binding. In addition, the authors specifically indicated that the *β4GalNT2* silencing gene strongly weakens human and nonhuman primate antibody binding. As a result a diminished porcine xenoantigenicity was observed (25). Another group that has taken advantage of CRISPR/Cas9 system (ear fibroblasts originated from pigs and were transfected with Cas9-GFP-GGTA1 plasmids throughout electroporation) to create Yucatan miniature pigs with triple knockout of the genes: *GGTA1*, cytidine monophosphate-N acetylneuraminic acid hydroxylase (*CMAH*), and alpha 1,3-galactosyltransferase 2 (*A3GALT2*) were Shim and colleagues (25). The binding between porcine PBMCs, aorta endothelial cells (AECs), cornea endothelial cells (CECs) and human IgM/IgG was assessed. Their cytotoxicity in human sera was also investigated. They paid particular attention to fact that the genetic alterations of donor pigs for xenotransplantation goals should be personalized to the target organ. The approach involving silencing extra genes such as CMAH or A3GALT2 may not always be necessary in Yucatan miniature pigs (26).

Another study (26) showed that pigs with the following genetic modifications: TKO.CD46.CD55.TBM.EPCR.HO-1.CD47 could be a reliable source of organs for humans. Nonetheless, noticeable growth of organs like pig kidneys and hearts during first few months counting from the xenotransplatation procedure performed can be still stimulated by growth hormone. Therefore, another approach involves knockout of the gene for growth hormone receptor is needed (27).

## 3 Review of organs derived from alpha-Gal deficient pigs

### 3.1 Bone-derived materials in xenotransplantation

The immunogenic protein components in the bone matrix do not occur in large quantities. As contrary vascularized organs such as kidney, heart, and liver are rich in immunogenic protein components. Bone tissue is the second most commonly transplanted biological material after blood. Thus, bone tissue from GT-KO pigs could serve as a promising graft material (28). Tseng et al. (28) examined the transplantation of bone marrow (BM) cells from miniature swine homozygous for GT-KO. A total of 10^8^ BM cells were infused into baboons. By using BM cells from GT-KO pigs, the chimerism and cellular hyporesponsiveness were detected. Nevertheless, stable engraftment and chimerism were not eventually reported (29).

Ezzelarab et al. (29) conducted interesting study. They examined the influence of alpha-Gal knock-out and CD46 knock-in on the human T-cell response to porcine mesenchymal stromal cells (pMSCs) *in vitro*. For this purpose, the authors isolated pMSCs from blood or bone marrow of WT, GT-KO, and GT-KO/CD46 pigs. The results indicated attenuated binding of primate antibody and T-cell response to GT-KO and GT-KO/CD46 pMSCs compared to those effects observed with WT pMSCs and to GT-KO pig aortic endothelial cells (pAEC). Collectively, GT-KO/CD46 may have a valuable immunomodulatory effect on the cellular response of primates to xenotransplant of pig cells or organs (30).

Kim et al. (30) examined the association between the absence of alpha-Gal epitope in bone tissue and suppressed production of inflammatory cytokine by human PBMCs *in vitro*. The PBMCs isolated from heparinized blood of healthy controls were induced with bone extracts of pigs with GT-KO. As a result, a reduction production of TNF-α, IL-2, IFN-γ, IL-17 and IL-1β as well as limited activation of CD4+ helper T cells was observed. The authors indicated that alpha-Gal KO pig bone xenografts could serve as an alternative to autografts and allografts (31).

Yamada et al. (31) published data showing that expression of human CD47 on porcine BM cells may prevent the deprivation of circulating porcine BM cells in nonhuman primates. Briefly, they depicted new strategy involving intra-bone marrow transplantation resulting in (i) a high percentage of long-term macro-chimerism (over 3 weeks) and (ii) a high incidence of BM transplants showing hyporesponsiveness to xenogeneic barriers in pig-to-baboon model (32).

Another group (32) demonstrated that GT-KO-pig cancellous bone has the ability to inhibit xenotransplant rejection and promote new bone formation in rhesus monkeys. GT-KO group reduced the ratio of CD4+ to CD8+ T cells and cytokines as among others IFN-γ and IL-2 which inhibited xenotransplant rejection. On the other hand, this group also observed production of osteoblastic markers like Runx2, OSX and OCN (33).

### 3.2 Kidney

Unmodified pig kidneys generate a strong innate immune response in primates within hours. Binding of natural antibodies to the porcine xenograft endothelium results in the activation of classical complement pathway and coagulation cascade. It results in congestion, edema, and massive interstitial hemorrhage. If immunosuppression is used to stop the T cell–mediated adaptive response, the survival of renal xenografts is extended by days or even weeks. After that second antibody-mediated process AHXR, also known as acute vascular rejection or delayed xenograft rejection may occur (34). Also, the non-alpha-Gal carbohydrate antigens like glycolylneuraminic acid (Neu5Gc; HD antigen) encoded by the cytidine monophospho-N acetylneuraminic acid hydroxylase (*cMAH*) gene and glycosyltransferase, (SD(a) antigen) encoded by the b-1,4-N acetyl-galactosaminyl transferase (*B4GalNT2*) gene are strongly connected with the effect of porcine kidney transplantation (35).

In kidney transplantation, expression of the human complement regulatory protein CD59 shows potential for prolonging the survival of transplanted organs *in vitro*. In turn, CD55 regulates complement activation, while CD46 acts as an inhibitory regulator of the complement system. Thrombomodulin and CD39 are also important factors, as they participated in complement activation and the coagulation cascade during heterogeneous immune regulation. Finally, an immunosuppressive regimen based on the blockade of the CD40-CD40L co-stimulation pathway is considered an essential step in renal xenotransplantation (36).

Wong et al. (36) using GT-KO target cells evaluated whether patients characterized by high anti-human panel reactive antibodies (PRA) are at increased risk for presensitization against inbred GT-KO miniature swine. For this purpose, they used sera from patients waiting for a kidney transplant from a deceased person. Anti-pig IgM/IgG antibody binding and complement-dependent cytotoxicity (CDC) assays, were notable diminished on GT-KO versus standard swine. There was no correlation between the degree of anti-human PRA and xenoreactivity to standard or GT-KO miniature swine. It was concluded that highly allosensitized patients awaiting kidney transplantation did not appear to be at no increased risk of xenosensitization compared with non-sensitized cohorts (37). Similar evidence was demonstrated by Hara et al. (37) who investigated the level and cytotoxicity of antibodies directed to non-Gal antigens on GT-KO pig PBMC in the serum samples derived from allosensitized patients awaiting for a kidney transplant. The obtained results indicate that although healthy volunteers tested produce cytotoxic antibodies to GT-KO PBMC, allosensitized patients will be at no greater risk of rejection of the porcine xenograft by a humoral mechanism (38).

An interesting study (38) demonstrated the usefulness of xenogenic thymokidney transplants taking advantage of a steroid-free immunosuppressive regimen and proved that the porcine thymus tissue (obtained from GT-KO miniature swine) stimulated early baboon thymopoiesis. It was correlated with donor-specific unresponsiveness *in vitro*. The average recipient survival of over 50 days was achieved (39).

Butler et al. (39) hypothesized that isoglobotrihexosylceramide synthase (iGb3s) coded by *A3GalT2* gene because of its capacity to synthesize isoglobo-series glycosphingolipids with an alpha-GAL-terminal disaccharide (iGb3) may provide alpha-Gal epitopes in *GGTA1* ^-^/^-^ animals. They targeted the *GGTA1* and *A3GalT2* genes in pigs using CRISPR/Cas9 system. Their data clearly indicate that *iGb3s* gene silencing notable modified the kidney’s glycosphingolipid profile. But the influence on alpha-Gal levels, antibody binding, cytotoxic profiles of baboon and human serum samples on porcine PBMCs remain unchanged. Hence, they concluded that *iGb3s* does not contribute to antibody-mediated rejection (AMR) in pig-to-primate or pig-to-human xenotransplantation (40).

An interesting study was carried out by Iwase et al. (40) which involved life-supporting kidney transplantation based on anti-CD40mAb-based regimen in baboons. Long-term survival was obtained in two baboons with kidneys from a transgenic pig (GT-KO/CD46/CD55/EPCR/TFPI/CD47). Moreover, the authors concluded that expression of human EPCR (+/− CD55) in the kidney may be important (41).

Ma et al. (2021) transplanted renal xenografts from pigs deprived of following crucial carbohydrate xenoantigens, *alpha-Gal*, *Neu5Gc*, and *SDa* (TKO) and expressing multiple human transgenes (hTGs) on the cells in cynomolgus monkeys. According to them, prolonged, rejection-free renal xenograft survival with TKO- hTG pigs transplanted in nonhuman primates was proved. Importantly, CD4+T cell depletion and low anti- pig antibody level were not absolutely needed for extended survival of TKO- hTG renal xenografts (42).

Montgomery et al. (42) transplanted kidneys from GT-KO pigs into two brain-dead human recipients. They proved that 54 hours after reperfusion, in two xenografts, a thoroughly intact architecture with preserved glomerular basement membrane and podocytes were detected. Hence, there was no sign of HAR. Nevertheless, the authors indicated that the serious drawback of their study was short follow-up because of the practical restrictions involving establishment of protocol in deceased people (43).

The first clinical-grade porcine kidney xenotransplant performed on a deceased human model was achieved by Porrett and group (43). They first carried out bilateral native nephrectomies in a human brain-dead decedent and then transplanted kidneys from genetically modified pigs. The pigs possessed ten genetic modifications including *among others* targeted insertion of two human complement inhibitor genes (hDAF, hCD46), two human anticoagulant genes (hTBM, hEPCR), and two immunomodulatory genes (hCD47, hHO1), as well as deletion of three pig carbohydrate antigens and the pig growth hormone receptor gene. No hyperacute rejection was reported after 72h. The Biopsies performed revealed thrombotic microangiopathy that did not developed and finally although the xenografts produced some urine, creatinine clearance was not observed (44).

A comprehensive research was done by Firl and Markmann (44). They analyzed and summarized 1051 non-human primate (NHP)- to- NHP or pig- to- NHP transplants mentioned in 88 articles (involving gene-edited donors containing at least knockout of alpha-1,3-galactosyltransferase). The authors concluded that preclinical renal allotransplantation survival in the NHP is significantly shorter than that of the well-established standard clinical allotransplantation. Additionally, it was demonstrated that genetic complement regulatory protein knock-in, as well as pharmacologic complement inhibitors regularly administered in the recipient reveal protective association for overall survival (45).

Preclinical data indicate that genetically modified pig kidney transplants in thoroughly selected, cross-match-negative human undergoing suppression with a CD40/CD154 co-stimulation pathway blockade-based regimen would probably function over a year (46).

Heo et al. (46) analyzed tissue samples from NHPs transplanted with organs of GT-KO transgenic pigs demonstrating expression of MCP or CD39. Authors intended to settle whether PERV is transmitted to host tissues after procedure. They demonstrated the lack of transmission of PERV in heart xenotransplant tissues. In turn, PERV-A, B, and C were noticeable in the NHP bladder after kidney xenotransplantation. Interestingly, PERV did not integrate into the host chromosome after kidney transplantation (47).

Another interesting study was done by Yang and collaborators (47). They used CRISPR/Cas9 gene editing technology, PiggyBac transposon and somatic cell nuclear transfer (SCNT) methods to construct four-gene-edited (GTKO/hCD55/hTBM/hCD39) *Diannan* miniature pigs. After that they executed kidney transplantation from pig to rhesus monkey to assess the efficacy of these porcine donors. The kidney xenograft survived for 11 days. The researchers reported about normal physiological and biochemical parameters. Importantly, they observed no hyperacute rejection or coagulation aberrations (48).

Wang et al. (48) successfully executed two pig-to-human kidney xenotransplants using genetically modified minipigs: with triple-gene knockouts (GGTA1, b4GalNT2, CMAH) and human gene transfers (hCD55 or hCD55/hTBM). They reported that renal xenograft functioned satisfactorily. Nevertheless, immunosuppression (T cell-mediated rejection and antibody-mediated rejection, confirmed by NK cell and macrophage infiltration) without blockade of CD40-CD154 pathway was unsuccessful in preventing acute rejection by day 12 (49).

Eisenson, et al. (49) were the first to prove the consistent survival in consecutive cases of pig-to-NHP kidney xenograft transplantation using source pigs with 10 genetic modifications. According to authors no other studies concerning solid organ pig-to-NHP transplantation led to xenograft survival longer than one month without CD40/CD154 costimulatory blockade. This blockade actually is not approved by the FDA. Authors showed long-term survival using FDA-approved immunosuppression (50). Judd and others (50) presented, for the first time, porcine kidney xenograft physiology in a human. Those results can be used to develop phase 1- based protocols in living persons. A deceased brain-dead adult underwent bilateral native nephrectomies followed by gene-edited (including 4 gene knockouts (GTKO, CMAH, B4GALNT2, and GHR) and 6 human transgenes (CD46, CD55, CD47, THBD, PROCR, and HMOX1) pig-to-human xenotransplantation. In a human decedent model, xenotransplantation of 10 gene-edited pig kidneys resulted in physiologic equilibrium for seven days (based on measured physiologic indicators such as levels of secreted renin, aldosterone and angiotensin II and parathyroid hormone) (51).

### 3.3 Heart

Over the past 30 years, orthotopic pig-to-NHP heart xenotransplantation has progressed significantly, with recipient survival increasing from just a few hours (1994) to several months (2024). It could be achieved thanks to scientific progress in donor genetics, organ preservation, immunosuppressive and immunomodulatory treatments, donor organ growth inhibition and prevention of porcine cytomegalovirus infection (52).

There is evidence (52) that GT-KO pigs increase the length of graft survival (2–6 months). Briefly, hearts from α1,3-galactosyltransferase knockout pigs were transplanted heterotically into baboons using an anti-CD154 monoclonal antibody–based regimen. However, eventually the development of thrombotic microangiopathy led to graft failure. The authors also concluded that levels of IgM or IgG against alpha-Gal even after all immunosuppressive therapy even antibodies below the detection threshold may still be induced (53).

Another study (53) proved that both alpha-Gal antigen and alpha-Gal antibodies play a key role in the calcification process of valvular bioprostheses. In glutaraldehyde-fixed pig pericardium pre-incubated with human anti-Gal antibodies, the increased calcification was observed in alpha-Gal-positive pig pericardium in comparison with GT-KO pig pericardium. Consequently, GT-KO porcine pericardium may serve as a new source of material for bioprosthetic heart valves (54).

Diswall et al. (54) compared sera from baboons transplanted with GT-KO hearts with human sera in relation to reactivity with pig glycolipids. Firstly, they proved that GT-KO heart and kidney deprived of alpha-Gal-terminated glycolipids entirely. Then, they demonstrated that baboon and human serum antibodies presented a distinct reactivity pattern to pig glycolipid antigens (particularly it involves acidic compounds). It clearly suggests that non-human primates have some drawbacks as a human pre-clinical model for immune rejection - based research (55).

Mohiuddin et al. (55) assumed that B-cells are the main cause of graft injury in baboon heart xenograft recipients even though anti-CD154 and mycophenolate mofetil (MMF)-based immunosuppression regimen is implemented. Thus, the authors used anti-CD20 antibody at the time of cardiac xenografts from GT-KO.hCD46Tg pigs to achieve significantly reduced level of circulating and secondary lymphoid B cells in baboons. This approach resulted in the inhibition of anti-pig immune response, graft injury, and reduced systemic coagulation pathway dysregulation (56).

Azimzadeh et al. (56) based on multi-center study comparing results of heart or kidney grafts from GT-KO pigs suggested that transgenic expression of a human complement pathway-regulatory protein (hCPRP) in the vascular endothelium of GT-KO pig (i) is correlated with the reduced risk of early graft failure (EGF), (ii) diminishes deposition and platelet activation which in turn correlates with EGF level as well as they indicated that (iii) although GT-KO.hCPRP pig reduces EGF, it does not eliminate systemic coagulation activation (57).

An interesting study was performed by McGregor and group (57) who demonstrated that GT-KO and alpha-Gal- positive porcine tissues had the same overall morphology and collagen content. Uniaxial stress and suture retention tests also showed that those tissues had comparable tensile strength. Based on that, authors concluded that knockout of the *GGTA1* does not affect structural integrity of porcine pericardium (58).

Abicht et al. (2017) conducted *ex vivo* perfusion of GT-KO/hCD46/HLA-E/hβ2-microglobulin transgenic pig hearts with human blood. Due to the fact that early cellular rejection reactions are mediated by NK cells and may be stopped by HLA- E, the authors hypothesized that transgenic GT-KO pigs expressing hCD46 and HLA- E may protect porcine grafts. They proved that tested combination of genetic modifications reduces damage caused by acute human- anti- pig rejection reactions *via* among others higher cardiac index in the first 2 hours of *ex vivo* perfusion and lower NK cell myocardial infiltration after perfusion (59).

Another study (59) described a 57-year-old man with nonischemic cardiomyopathy who was not a candidate for standard therapeutics and therefore he received a heart from a 10-gene-edit pig donor (Revivicor) in combination with anti-CD40 monoclonal antibody (Kiniksa Pharmaceuticals), and an XVIVO heart perfusion system (XVIVO Perfusion). This led to the patient’s support for 7 weeks. Unfortunately, on day 49 after transplantation, abrupt diastolic thickening and xenograft failure was notable (60).

Mohiuddin, et al. (60) reported orthotopic (life-sustaining) survival of genetically modified porcine heart xenografts (with six gene modifications) for nearly 9 months in baboon recipients. The baboons were transplanted with life-supporting xenografts containing multiple human complement regulatory, thromboregulatory, and anti-inflammatory proteins, in addition to growth hormone receptor (GHR) knockout (KO) and carbohydrate antigen KOs. Some “multi-gene” xenografts have shown survival longer than 8 months without the need for supportive medications and with no signs of abnormal xenograft thickness or rejection (61).

According to Moazami, et al. (62) a genetically modified porcine heart xenotransplantation was carried out in a non-ambulatory patient with end-stage heart failure undergoing extracorporeal membrane oxygenation support and who was found to be ineligible for allograft transplantation. Hyperacute rejection was avoided. Unfortunately, the recipient rapidly developed diastolic dysfunction and global pathologic thickening of the myocardium within the xenograft. Possible etiologies of xenograft endothelial cell damage can be distinguished: 1) endogenous xenoantibody-mediated rejection, 2) exogenous administration of IVIG-containing xenoantibodies, and 3) porcine cytomegalovirus/porcine roseolovirus reactivation within the xenograft. The authors concluded that the GE pig heart and anti-CD40-based regimen could keep the patient alive for 60 days (62). In another study, Moazami, et al. (62) taking advantage of 10-gene-edited pigs, transplanted hearts into two brain-dead human recipients. They focused on monitoring xenograft function, hemodynamics and systemic responses for 66 hours. Immediately after transplantation both xenografts demonstrated satisfying cardiac function. But cardiac function declined postoperatively in one case. Furthermore, there is no evidence of transmission of zoonoses from the donor pigs to the human recipients. Those results indicated that pig-to-human heart xenotransplantation can be achieved successfully without evidence of hyperacute rejection or zoonosis (63). Singh et al. (63) encouraged by previous accomplishment: 9-month survival of hearts with seven genetic modifications transplanted in baboons, they also demonstrated successful transplantation of 10-GE pig hearts with GT-KO and overexpression of human genes to prevent rejection in non-human primates. Those ten gene-edited cardiac xenografts involved deletion of 4 genes (i.e., *GGTA*), SDa blood group antigen (*B4GALNT2*), and N-glycolylneuraminicacid (*CMAH*) and growth hormonereceptor (*GHR*)) and overexpression of six human genes (h*CD46*, h*DAF*, h*TBM*, h*EPCR*, h*CD47* and h*HO-1*) as well as provided life-supporting function up to 225 days in a non-human primate model (64).

Griffith and colleagues (64) transplanted a ten gene-edited pig heart into a 58-year-old man with progressive, debilitating inotrope-dependent heart failure caused by ischemic cardiomyopathy which excluded utilizing standard therapies for advanced heart failure. The patient was maintained on a costimulation (anti-CD40L, Tegoprubart) blockade-based immunomodulatory regimen. The xenograft functioned well for the first few weeks. Subsequently, rapidly progressing diastolic heart failure, biventricular wall thickening and, ultimately, near-complete loss of systolic function occurred, necessitating the initiation of extracorporeal membranous oxygenation. Thus, new approach to avoid antibody-mediated graft rejection are strongly required (65).

### 3.4 Lungs

Lung xenotransplantation faces ongoing challenges due to the lung’s sensitivity to injury and its complex immune rejection mechanisms. Rapid coagulation disorders are a significant barrier in lung transplantation. Consequently, additional strategies to manage coagulation dysregulation in lung xenotransplantation should be prioritized (66, 67). Notably, pig lungs contain unique immune cells, *inter alia* pulmonary intravascular macrophages which together with alveolar macrophages and non-T-cell leukocytes take part in recognizing and responding to pathogens in the respiratory tract (68).

An important study was conducted by Westall et al. (68). They studied *ex vivo* functional properties of lungs from genetically modified pigs: *i)* GT-KO, *ii)* GT-KO revealing simultaneously expression of the human complementary regulatory proteins CD55 and CD59 (GT-KO/CD55-59); and *(iii)* GT-KO demonstrating expression of both CD55–59 and CD39 (GT-KO/CD55-59/CD39). The GT-KO modification and overexpression of CD55-59 led to similar xenograft efficiency as with the single GT-KO modification. Although long-term lung function was observed in the genetically modified lungs, pathological changes consistent with intravascular thrombosis, platelet deposition and coagulation were ultimately confirmed. Nevertheless, the histological changes were less evident in the GT-KO/CD55-59/CD39 lungs in comparison with the other analyzed specimens (69).

Platz et al. (69) established a detergent-based protocol for the decellularization of wild-type and GT-KO pig lungs. According to them no obvious differences in histologic structure were observed. However, a 25% difference in residual protein was observed between decellularized lungs from wild-type and GT-KO pigs. It also concerned retention of alpha-galactosylated epitopes in acellular wild-type pig lungs. Moreover, an approach involving seeding alginate-coated decellularized lungs showed no significant difference in recellularization (70).

In the study performed by Stahl et al. (70) lungs derived from unmodified or GT-KO pigs were decellularized and subcutaneously implanted into a rhesus macaque model to assess the host immune response. Sham injury, native porcine lung, and allogeneic decellularized macaque lung served as control groups. The knockout of the alpha-Gal epitope in porcine lung tissue leads to delayed immune cell infiltration and reduces the chronic T-cell mediated reaction against decellularized components after re-implantation. These results highlighted differing immune cell profiles of circulating and infiltrating immune cells depending on the source of the implanted tissue and processing method used (71).

Watanabe et al. (71) tested survival of lung xenograft following IBBMTx in a pig-to-baboon model. For this reason, GalTKO-hCD47/hCD55Tg or -hCD55Tg or -hCD46/HLA-E Tg pig IBBMTx were transplanted into baboons. After 1–3 months, those baboons received lung xenografts from either hCD47+ or hCD47-porcine lungs. This study proved durable macrochimerism beyond 8 weeks and B cell tolerance in large animal xenotransplantation. hCD47Tg pigs as a source of IBBMTx and lung donors improves engraftment survival (72).

Gasek et al. (72) described in detail immune response to native and decellularized wild-type and GT-KO pig lungs. Those results indicate that decellularization process diminishes key immune recognition mechanisms involved in post-transplant survival, immunoglobulin reactivity and complement activation. Importantly, no significant immune advantage was observed in in the lungs from GT-KO pigs regarding macrophage phenotype or phagocytosis (73).

Burdorf et al. (2021) assessed the immunological response induced in a baboon *in vivo* lung xenotransplant model. The authors investigated lungs from genetically modified pigs based on physiological incompatibilities between pigs and humans. For this purpose an expression of human complement and coagulation pathway regulatory proteins, anti-inflammatory enzymes and self-recognition receptors as well as knock-down of the *β4Gal* xenoantigen were applied and analyzed in different combinations. Transient life- sustaining GalTKO.hCD46 lung function was shown in connection with human thrombomodulin (hTBM) or endothelial protein C receptor (hEPCR) (74).

Chaban et al. (74) pointed out that expression of human complement pathway regulatory members like CD46 or CD55 helps to improve survival of pig organ xenografts. To confirm this hypothesis, they used GT-KO lungs heterozygous for human *CD46* (GT-KO.heteroCD46), lungs homozygous for *hCD46* (GT-KO.homoCD46), and GT-KO.homoCD46 lungs also heterozygous for *hCD55* (GT-KO.homoCD46.hCD55) which were subsequently perfused with human blood in an *ex vivo* circuit. They revealed that elevated *hCD46* expression contributed to the significantly prolonged lung survival significantly but surprisingly did not reduce complement factor C3a levels (75).

### 3.5 Cornea

Due to its avascular nature, porcine cornea exhibits lower alpha-Gal expression. This leads to reduced immunologic recognition of antigens and a great chance for protection against HAR. Therefore, porcine cornea appears to be an ideal substitute for the human cornea. However, this immune privilege can be easily disrupted by e.g. prior infections or inflammations in human recipient corneal beds. It is important to bear in mind that if a porcine corneal graft is transplanted into a vascularized corneal bed, xenogeneic antigens are available to pre-existing donor pig-specific antibodies like anti-alpha-Gal antibodies. While corneal tissue benefits from relative immune privilege, GT-KO modification alone may be insufficient in high-risk recipients without additional immunosuppression (76).

Hara et al. (76) analyzed the *in vitro* human humoral and cellular immune responses of wild-type pig corneal endothelial cells (pCECs) and pig aortic endothelial cells (pAECs) These processes were then compared with CECs from GT-KO/CD46 pigs and human donors The obtained results showed that the human humoral and cellular immune responses to genetically modified pCECs were significantly inhibited compared with those to wild-type pCECs, but were not characterized by a such low immunogenicity as observed in human CECs (hCECs). Among others, the authors demonstrated inferior expression of SLA class II on the pCECs compared with that on the pAECs (77).

In another study (77) the relationship between decellularization process and the reduction of the immunogenicity of pig corneas were investigated. The authors showed that although α-Gal affects long-term graft survival of porcine corneal xenografts, it does not impact acute rejection. The cultured porcine corneal endothelial cells (pCECs) on the surface of decellularized corneal tissue formed a monolayer as in a native cornea demonstrating the utility of decellularized cornea as a suitable scaffold for CECs. Consequently, the authors concluded that the decellularized porcine corneas may contribute to the long-term survival of porcine corneal xenografts (78).

According to Lee et al. (78) the lack of alpha-Gal and N-glycolylneuraminic (Neu5Gc) expression on the porcine cornea and aorta fits into approach: from bench to bedside. They revealed that the use of corneal xenografts from pigs deficient in both alpha-Gal and Neu5Gc in humans is strongly correlated with the reduced human xenoreactive antibody binding and thus with the attenuated immunologic and/or inflammatory injury. Nonetheless, it will not prevent all antibody binding. They revealed that the level of human IgM/IgG binding was significantly reduced in the case of absence of Neu5Gc on GT-KO aortic tissue and aortic endothelial cells. However, there was no noteworthy difference in binding of IgM/IgG between GT-KO and GT-KO/Neu5Gc KO corneal endothelial cells (79).

Dong et al. (2018) compared properties of full- thickness corneal xenografts from wild-type and GT-KO pigs with the additional expression of a human complement regulatory protein (GT-KO/CD46 pigs) in rhesus monkeys. The substantial difference in graft survival between wild-type and GT-KO pig corneas was not observed. They hypothesized that sensitization against non-alpha-Gal antigens could not be avoided by local steroid injections and therefore local and immunosuppressive therapy may be required to overcome inflammation and an immune response following full- thickness corneal xenotransplantation (80).

Yoon et al. (80) conducted experiment involving full‐thickness corneal xenotransplantation in rhesus macaques using GT-KO miniature (GT-KOm) pigs with or without anti CD20 Ab treatment. The aim was to assess the effect of porcine GT-KOm-derived grafts on graft survival duration. Graft survival was prolonged in the CD20 group than control in group. GT-KOm pig corneas constitute a promising alternative for human transplantation but this approach requires an appropriate immunosuppression like aforementioned anti‐CD20 Ab treatment. GT-KO modification alone is not sufficient to exclude rejection, hence inhibition of B cells and complement activation is essential (81).

### 3.6 GT-KO cells & cell lines

Kim et al. (81) designed knock-in vectors for expression of human decay-accelerating factor (*hDAF*) gene on the *GGTA1 locus* and then isolated heterozygous porcine somatic cells transfected with this knock-in vector. The survival rate of heterozygous cells exposed to human serum was noticeable higher than that of control and GGTA1 knock-out heterozygous cells. Consequently, GGTA1 knock-out cell lines expressing DAF from porcine ear fibroblasts for SCNT were established. This approach may potentially ensure an unlimited source of transgenic pigs for xenotransplantation purposes (82).

An interesting research was conducted by Liu et al. (82). They generated pig induced pluripotent stem cells (piPSCs) from GT-KO tissue *via* overexpression of *POU5F1*, *SOX2*, *NANOG*, *LIN28*, *KLF-4*, and *C-MYC* reprogramming genes. These GT-KO piPSCs revealed characteristics of iPSCs such as expression of *SSEA1* and *SSEA4* as well as high telomerase activity (83).

Kumar et al. (83) characterized and evaluated functionality of adipose mesenchymal stromal cells (AdMSCs) from GT-KO transgenic for the human complement-regulatory protein CD46. Interestingly, the proliferative capacity of hPBMC to GT-KO/hCD46 pAdMSC and hAdMSC stimulators were lower than to GT-KO pAEC. The proliferation rate of hPBMC to GT-KO pAEC was diminished by GT-KO/hCD46 pAdMSC and hAdMSC. However, the supernatant collected from GT-KO/hCD46 pAdMSC did not inhibit the human cell – cell contact-dependent xenoresponse to GT-KO pAEC. It emphasizes the fact that genetically engineered pAdMSC function across the xenogeneic barrier and may be crucial in cellular xenotransplantation (84).

Another group (84) assessed the ability of pig adipose mesenchymal stem cells (AMSCs) to osteogenesis *in vivo* in a nude rat model. A significant drop of anti-pig IgG (at 1 month) in rats implanted with GT-KO AMSCs in contrast to those implanted with AMSCs rich in alpha-Gal epitope was observed. Additionally, lymphocyte and macrophage infiltration of xenografts consisting of pig AMSCs after osteogenic differentiation was noticeably lower in recipients of GT-KO pig cells. It suggests that the cellular immunomodulation with the use of GT-KO AMSCs and the significant improvement of the cellular engraftment of pig osteogenic cells by delaying xenorejection can be achieved (85).

As reported by Lee et al. (85) porcine tissues (aortas, corneas) and cells (RBCs, PBMCs, and AECs) lacking Neu5Gc expression showed significantly reduced human antibody binding. On the contrary, CECs were not correlated with the reduced human antibody binding. The authors used material isolated from GT-KO/hCD46 and GT-KO/hCD46/Neu5Gc KO pigs. However, the lack of Neu5Gc expression on GT-KO/hCD46 pAECs did not reduce human platelet aggregation, nor direct blood-mediated inflammatory response to pig islets (86).

Bongoni et al. (86) hypothesized that Corline Heparin Conjugate (CHC) - a compound of numerous unfractionated heparin chains which covers cells with a glycocalyx-like layer may reduce induction of the plasma cascade systems after xenotransplantation. They studied protective properties on pig AECs from wild-type and genetically modified (GT-KO. hCD46.hTBM) pig. As a result, they indicated that CHC coating and genetic modification contribute to strong compatibility with human blood, pointing out that pre-transplant perfusion of genetically engineered pig organs with CHC may have a desirable impact on post-transplant xenograft function (87).

The available data (87) show that stable transgenic expression of human thrombomodulin (hTBM) in pig endothelial cells plays a crucial role in regulating inflammation-mediated coagulation dysregulation response after pig organ xenotransplantation in primates. AECs derived from GT-KO/CD46 and GT-KO/CD46/hTBM pigs were stimulated *via* hTNF-α and the level of the inflammatory/coagulation regulatory protein was examined. After hTNF-α stimulation, the evident expression reduction of inflammatory molecules on GT-KO/CD46/hTBM pAECs in comparison with GT-KO/CD46 pAECs was observed (88).

Interesting results were reported by Huai et al. (88). They indicated that *TKO/hCD55/hTM/hEPCR* six gene-edited pig may be an ideal candidate for xenotransplantation purposes. It was proved that high expression of hCD55, together with the co-expression of the *hEPCR* and *hTM* genes successfully reduced the human complement cytotoxicity and improved anticoagulant capacity in transgenic pigs. These six gene-edited pigs may reveal great compatibility with humans but importantly with minimal gene combinations. For this research, they used isolated pAECs from six gene-edited pigs and checked IgM and IgG binding, complement cytotoxicity, and thrombin-antithrombin (TAT) complex degree (89).

## 4 Future directions and limitations of GT-KO pig-based xenotransplantation

The rapid progress in genetic modifications contributes to the development of xenotransplantation (**Table 1**). However, it is important to bear in mind that although genetic modifications may overcome innate responses, genetic engineering is not satisfactory to prevent long-term rejection. Thus, additional strategies like targeted immunosuppression or tolerance induction of B and T cells will be essential for extended survival of engraftments (90).

**Table 1:** The summary of progress made in the field of xenotransplantation using components from genetically modified pigs.

Organ/cell line	Current state of knowledge	References
1. Bone-derived materials	• Promising graft material due to the fact that immunogenic protein components in the bone matrix are scarce • The following genetically engineering pig-derived components were successfully obtained: mesenchymal stromal cells, peripheral blood mononuclear cells, bone marrow, bone	(28–33)
2. Kidney	• Numerous clinical trials documented (including brain-dead decedent model) • 10 gene-edited pig kidneys are now intensively investigated	(34–51)
3. Heart	• pig-to-NHP heart xenotransplantation is developed for about 30 years • A ten gene-edited pig heart was transplanted into a patient with progressive, debilitating inotrope-dependent heart failure • In a non-human primate model ten gene-edited pig heart provided life-supporting function up to 225 days.	(52–65)
4. Lungs	• Challenging due to lung’s complex immune rejection mechanisms • In a non-human primate model lung’s targeted genetic modifications and pharmacological treatments resulted in an extended survival	(66–75)
5. Cornea	• Porcine cornea exhibits low expression of alpha-Gal • In rhesus monkeys GT-KO graft survival was significantly prolonged but still insufficient	(76–81)
6. Cells/cell lines	• Six gene-edited pig aortic endothelial cells may constitute a minimal gene combinations to achieve maximum compatibility with human body.	(82–89)

Alpha-Gal is one of three key antigens of importance in xenotransplantation (particularly in cardiac surgery), and its immunogenicity in humans is well described. Some methods, such as decellularization process and glutaraldehyde fixation can reduce the immune response against bioprosthetic valves, but eventually they do not eliminate it. Consequently, patients receiving bioprosthetic valves are characterized by an elevated level of alpha-Gal IgG and IgM leading to valve degradation over time (6). An important unresolved question is whether alpha-Gal can be completely eliminated from pig cells. Based on available literature data, GT-KO pigs still express alpha-Gal but this is less than about 2% of the level of wild-type pigs. This may be explained by the fact that probably another glycosyltransferase synthetizes alpha-Gal. Whether this influences graft rejection remains questionable and requires further study. The generation of genetically engineered pigs that are deprived of alpha-Gal epitope has been a big achievement in the development xenotransplantation (91).

However, these organ-source pigs do not solve all occurring immunologic problems. Potential contributing factors are associated with i) the activity of preformed anti-nonGal immunoglobulins or ii) low levels of produced antibodies to nonGal antigens, iii) NK cell or macrophage influence, iv) and cogenital coagulation dysregulation between pigs and primates. Thus, much research is focusing on detailed analysis of porcine antigen targets for human preformed anti-nonGal antibodies and creating GT-KO pigs that are transgenic for one or more human “anticoagulant” genes (92). Due to this fact, the simultaneous strategies to reduce alpha-Gal epitope are strongly investigated. This includes, among other approaches like expressing a gene encoding human H transferase by porcine cells. This enzyme has the ability to catalyze the addition of fucose using the identical receptor (N-acetyllactosamine) as α1,3-galactosyltransferase. Consequently, activity of these enzymes is based on competition. A co-expression of a1,2-fucosyltransferase and another enzyme - α-galactosidase seems to be promising. It allows the enzymatic removal of terminal D-galactose residues from the cell surface (93).

Organs from triple-knockout (TKO) pigs may alone be sufficient during first clinical trials, but the allograft may be at risk from complement injury correlated with ischemia-reperfusion or a systemic infection. Therefore, it would seem reasonable to use graft expressing human complement-regulatory proteins like CD46, CD55, CD59. Those proteins are suitable for defending a porcine engraftments against the effects of human complement (94). Zhang et al. (94) selected 32 genes that are not found in the human genome (among others: *PLEKHS1*, *TOM1L1*, *MCCD1*, *MUC4*, *PKP1*, *KLHDC7A*, *SFRP5*, *CYP24A1*, *SEMA4D*, *ESRRG*, *LY75*, *TM4SF4*, *TBXAS1*, *FOXJ1*, *HOXD1*, *FBXO2*, *PLLP*, *KCNJ5*, *IQGAP2*) which might be main immunologic targets involved in delayed xenograft rejection (DXR). They could be knocked out as well as the immunosuppressive therapy could be applied to prevent the organism’s response to expression of those genes (95). A strategy involving genetic engineering to protect the grafts from the activation of the human adaptive immune response may involve deprivation of SLA class 1, downregulation of SLA class II or expression of PD-L1. Expectantly, it will enable reduced exogenous immunosuppressive therapy (96).

Nonetheless, overexpression of too many protective genes is not always a good idea due to the risk of redundant genetic elements. Those elements could hypothetically influences the health of pig or the function of a specific donor organ. Besides, the possible synergies and shared drawbacks connected with the inserted multiple genes is not well understood and more data are needed to better elucidate the key aspects of the clinical consequences of xenotransplantation with the use of pig models with various combinations of multiple genetic modifications (97).

It is also important to assess the risk of cross-species infection (xenozoonosis) when xenotransplantation of solid organs is considered. Particularly porcine endogenous retroviruses (PERV) seem to be a real threat. Based on the receptor recognition three classes of PERV can be distinguished: PERV-A, PERV-B and PERV-C. PERV-A, PERV-B and recombinant forms of PERV-A/C are closely related with the safety level in clinical xenotransplantation (99).

## 5 Conclusions

The main aim of the present review was to evaluate various approaches to obtaining organs from genetically modified pigs that may be helpful in xenotransplantation. As noted above, primates produce specific antibodies against alpha-Gal epitope. Alpha-Gal is one of the key antigens in xenotransplantation. It is noteworthy that GT-KO pigs still express alpha-Gal at a minimum level because other enzymes besides alpha-1,3 galactosyltransferase may also synthesize alpha-Gal. Thus, multiple strategies aimed at reducing alpha-Gal epitope expression are actively under investigation. It would seem reasonable to also use a graft expressing human complement-regulatory proteins CD46, CD55, CD59 and many others. These proteins are protect pig graft against the effects of human complement. A major concern regarding the overexpression of an excessive number of protective genes is that it may pose risks due to redundant genetic elements. Those elements could potentially influence the health of pig or the function of a specific grafts. It is worth noting that although genetic modifications may overcome innate responses, genetic engineering alone is not enough to prevent rejection. Thus, additional strategies such as targeted immunosuppression or tolerance activation of B and T cells will be essential for prolonged survival of xenografts. However, more research is needed to resolve the many existing discrepancies regarding the development of transgenic pigs that would be ideal for clinical applications.

## References

[1] Ekser B Cooper DK. Update: cardiac xenotransplantation. Curr Opin Organ Transpl. (2008) 13:531–5. doi: 10.1097/MOT.0b013e32830fdf89, PMID: 19060538 PMC2652701

[2] Wennberg L Song Z Bennet W Sandberg JO Sundberg B Thall A. Importance of the Gal α1–3 Gal antigen in discordant islet xenotransplantation: immunosuppression, which inhibits porcine islet xenograft rejection in ordinary mice, is equally effective in Gal-knockout mice. Transplantation. (2004) 77:1275–80. doi: 10.1097/01.TP.0000119162.11743.AF, PMID: 15114098

[3] Li Q Lan P. Xenotransplantation: current challenges and emerging solutions. Cell Transpl. (2023) 32:9636897221148771. doi: 10.1177/09636897221148771, PMID: 36644844 PMC9846288

[4] Cooper DK Ezzelarab MB Hara H Iwase H Lee W Wijkstrom M. The pathobiology of pig-to-primate xenotransplantation: a historical review. Xenotransplantation. (2016) 23:83–105. doi: 10.1111/xen.12219, PMID: 26813438

[5] Martín-Lázaro J Núñez-Orjales R González-Guzmán LA González MT Boquete M Carballada F. Galactose-α-1,3-galactose (alpha-gal) allergy: first pediatric case in a series of patients in Spain. Allergol Immunopathol (Madr.). (2019) 48:251–8. doi: 10.1016/j.aller.2019.07.004, PMID: 31718865

[6] Zhong R. Gal knockout and beyond. Am J Transpl. (2007) 7:5–11. doi: 10.1111/j.1600-6143.2006.01615.x, PMID: 17227553

[7] Habiro K Syker S Yang YG. Induction of human T-cell tolerance to pig xenoantigens via thymus transplantation in mice with an established human immune system. Am J Transpl. (2009) 9:1324–9. doi: 10.1111/j.1600-6143.2009.02646.x, PMID: 19459808 PMC2752337

[8] Galili U. α1,3Galactosyltransferase knockout pigs produce the natural anti-Gal antibody and simulate the evolutionary appearance of this antibody in primates. Xenotransplantation. (2013) 20:267–76. doi: 10.1111/xen.12051, PMID: 23968556

[9] Hilger C Fischer J Wölbing F Biedermann T. Role and mechanism of galactose-alpha-1,3-galactose in the elicitation of delayed anaphylactic reactions to red meat. Curr Allergy Asthma Rep. (2019) 19:3. doi: 10.1007/s11882-019-0835-9, PMID: 30673913 PMC6344609

[10] Macdougall JD Thomas KO Iweala OI. The meat of the matter: understanding and managing alpha-gal syndrome. Immunotargets Ther. (2022) 11:37–54. doi: 10.2147/ITT.S276872, PMID: 36134173 PMC9484563

[11] Boksa M Zeyland J Slomski R Lipinski D. Immune modulation in xenotransplantation. Arch Immunol Ther Exp (Warsz). (2015) 63:181–92. doi: 10.1007/s00005-014-0317-7, PMID: 25354539 PMC4429136

[12] Miland J Christiansen D Lazarus BD Taylor SG Xing PX Sandrin MS. The molecular basis for galalpha(1,3)gal expression in animals with a deletion of the alpha1,3galactosyltransferase gene. J Immunol. (2006) 176:2448–54. doi: 10.4049/jimmunol.176.4.2448, PMID: 16456004

[13] Xuan Y Petersen B Liu P. Human and pig pluripotent stem cells: from cellular products to organogenesis and beyond. Cells. (2023) 12:2075. doi: 10.3390/cells12162075, PMID: 37626885 PMC10453631

[14] Ekser B Burlak C Waldman JP Lutz AJ Paris LL Veroux M. Immunobiology of liver xenotransplantation. Expert Rev Clin Immunol. (2012) 8:621–34. doi: 10.1586/eci.12.56, PMID: 23078060 PMC3774271

[15] Hryhorowicz M Zeyland J Slomski R Lipinski D. Genetically modified pigs as organ donors for xenotransplantation. Mol Biotechnol. (2017) 59:435–44. doi: 10.1007/s12033-017-0024-9, PMID: 28698981 PMC5617878

[16] Hryhorowicz M Lipinski D Hryhorowicz S Nowak-Terpilowska A Ryczek N Zeyland J. Application of genetically engineered pigs in biomedical research. Genes (Basel). (2020) 11:670. doi: 10.3390/genes11060670, PMID: 32575461 PMC7349405

[17] Kimsa-Dudek M Strzalka-Mrozik B Kimsa WM Blecharz I Gola J Skowronek B. Screening pigs for xenotransplantation: expression of porcine endogenous retroviruses in transgenic pig skin. Transgenic Res. (2015) 24:529–36. doi: 10.1007/s11248-015-9871-y, PMID: 25812516

[18] Ramsoondar J Zoltán M Cristina C Barry LW William LF Kenneth RB. Production of alpha 1,3-galactosyltransferase-knockout cloned pigs expressing human alpha 1,2-fucosylosyltransferase. Biol Reprod. (2003) 69:437–45. doi: 10.1095/biolreprod.102.014647, PMID: 12672664

[19] Phelps CJ Koike C Vaught TD Boone J Wells KD Shu-Hung C. Production of alpha 1,3-galactosyltransferase-deficient pigs. Science. (2003) 299:411–4. doi: 10.1126/science.1078942, PMID: 12493821 PMC3154759

[20] Dor FMJ Tseng YL Cheng J Moran K Sanderson TM Lancos CJ. alpha1,3-Galactosyltransferase gene-knockout miniature swine produce natural cytotoxic anti-Gal antibodies. Transplantation. (2004) 78:15–20. doi: 10.1097/01.tp.0000130487.68051.eb, PMID: 15257033

[21] Kolber-Simonds D Lai L Watt SR Denaro M Arn S Augenstein ML. Production of alpha-1,3-galactosyltransferase null pigs by means of nuclear transfer with fibroblasts bearing loss of heterozygosity mutations. Proc Natl Acad Sci U S A. (2004) 101:7335–40. doi: 10.1073/pnas.0307819101, PMID: 15123792 PMC409919

[22] Kwon DJ Kim DH Hwang IS Kim DE Kim HJ Kim JS. Generation of α-1,3-galactosyltransferase knocked-out transgenic cloned pigs with knocked-in five human genes. Transgenic Res. (2017) 26:153–63. doi: 10.1007/s11248-016-9979-8, PMID: 27554374 PMC5243873

[23] Wang RG Ruan M Zhang RJ Chen L Li XX Fang B. Antigenicity of tissues and organs from GGTA1/CMAH/β4GalNT2 triple gene knockout pigs. J BioMed Res. (2018) 33:235–43. doi: 10.7555/JBR.32.20180018, PMID: 30007952 PMC6813527

[24] Estrada JL Martens G Li P Adams A Newell KA Ford ML. Evaluation of human and non-human primate antibody binding to pig cells lacking GGTA1/CMAH/β4GalNT2 genes. Xenotransplantation. (2015) 22:194–202. doi: 10.1111/xen.12161, PMID: 25728481 PMC4464961

[25] Shim J Ko N Kim HJ Lee Y Lee JW Jin DI. Human immune reactivity of GGTA1/CMAH/A3GALT2 triple knockout Yucatan miniature pigs. Transgenic Res. (2021) 30:619–34. doi: 10.1007/s11248-021-00271-w, PMID: 34232440 PMC8478729

[26] Cooper DKC. The long and winding road to clinical xenotransplantation: A personal journey. Eur Surg Res. (2022) 63:165–72. doi: 10.1159/000525757, PMID: 35764060 PMC10124763

[27] Lu T Yang B Wang R Qin C. Xenotransplantation: current status in preclinical research. Front Immunol. (2020) 10:3060. doi: 10.3389/fimmu.2019.03060, PMID: 32038617 PMC6989439

[28] Tseng YL Dor FJM Kuwaki DR Ryan D Wood J Denaro M. Bone marrow transplantation from alpha1,3-galactosyltransferase gene-knockout pigs in baboons. Xenotransplantation. (2004) 11:361–70. doi: 10.1111/j.1399-3089.2004.00151, PMID: 15196131

[29] Ezzelarab M Ezzelarab C Wilhite T Kumar G Hara H Ayares D. Genetically-modified pig mesenchymal stromal cells: xenoantigenicity and effect on human T-cell xenoresponses. Xenotransplantation. (2011) 18:183–95. doi: 10.1111/j.1399-3089.2011.00635.x, PMID: 21696448

[30] Kim SE Kang KW Gu S Hwang S Ock SA Shim KM. Immunological compatibility of bone tissues from alpha-1,3-galactosyltransferase knockout pig for xenotransplantation. BioMed Res Int. (2018) 2018:1597531. doi: 10.1155/2018/1597531, PMID: 29967767 PMC6008681

[31] Yamada K Ariyoshi Y Pomposelli T Takeuchi K. Intra-bone bone marrow transplantation in pig-to-nonhuman primates for the induction of tolerance across xenogeneic barriers. Methods Mol Biol. (2020) 2110:213–25. doi: 10.1007/978-1-0716-0255-3_14, PMID: 32002911 PMC7412596

[32] Wang W Lu J Song Y Zeng C Wang Y Yang C. Repair of bone defects in rhesus monkeys with α1,3-galactosyltransferase-knockout pig cancellous bone. Front Bioeng Biotechnol. (2022) 10:990769. doi: 10.3389/fbioe.2022.990769, PMID: 36172016 PMC9510634

[33] Cowan PJ Cooper DKC d’Apice AJ. Kidney xenotransplantation. Kidney Int. (2014) 85:265–75. doi: 10.1038/ki.2013.381, PMID: 24088952 PMC3946635

[34] Kirkeby S Mikkelsen HB. Distribution of the alphaGal- and the non-alphaGal T-antigens in the pig kidney: potential targets for rejection in pig-to-man xenotransplantation. Immunol Cell Biol. (2008) 86:363–71. doi: 10.1038/icb.2008.1, PMID: 18301385

[35] Xu H He X. Developments in kidney xenotransplantation. Front Immunol. (2024) 14:1242478. doi: 10.3389/fimmu.2023.1242478, PMID: 38274798 PMC10808336

[36] Wong BS Yamada Okumi M Weiner J O’Malley P Tseng YL. Allosensitization does not increase the risk of xenoreactivity to alpha1,3-galactosyltransferase gene-knockout miniature swine in patients on transplantation waiting lists. Transplantation. (2006) 82:314–9. doi: 10.1097/01.tp.0000228907.12073.0b, PMID: 16906027

[37] Hara H Ezzelarab M Rood PPM Lin YJ Busch J Ibrahim Z. Allosensitized humans are at no greater risk of humoral rejection of GT-KO pig organs than other humans. Xenotransplantation. (2006) 13:357–65. doi: 10.1111/j.1399-3089.2006.00319.x, PMID: 16768729

[38] Griesemer AD Hirakata A Shimizu A Moran S Tena A Iwaki H. Results of gal-knockout porcine thymokidney xenografts. Am J Transpl. (2009) 9:2669–78. doi: 10.1111/j.1600-6143.2009.02849.x, PMID: 19845583 PMC2801602

[39] Butler JR Skill NJ Priestman DL Platt FM Li P Estrada JL. Silencing the porcine iGb3s gene does not affect Galα3Gal levels or measures of anticipated pig-to-human and pig-to-primate acute rejection. Xenotransplantation. (2016) 23:106–16. doi: 10.1111/xen.12217, PMID: 27106872

[40] Iwase H Hara H Ezzelarab M Li T Zhang Z Gao B. Immunological and physiological observations in baboons with life-supporting genetically engineered pig kidney grafts. Xenotransplantation. (2017) 24. doi: 10.1111/xen.12293, PMID: 28303661 PMC5397334

[41] Ma D Hirose T Lassiter G Sasaki H Rosales I Coe TM. Kidney transplantation from triple-knockout pigs expressing multiple human proteins in cynomolgus macaques. Am J Transpl. (2022) 22:46–57. doi: 10.1111/ajt.16780, PMID: 34331749 PMC9291868

[42] Montgomery RA Stern JM Lonze BE Tatapudi VS Mangiola M Wu M. Results of two cases of pig-to-human kidney. N Engl J Med. (2022) 386:1889–98. doi: 10.1056/NEJMoa2120238, PMID: 35584156

[43] Porrett PM Orandi BJ Jumar V Houp J Anderson D Killian AC. First clinical-grade porcine kidney xenotransplant using a human decedent model. Am J Transpl. (2022) 2:1037–53. doi: 10.1111/ajt.16930, PMID: 35049121

[44] Firl DJ Markmann JF. Measuring success in pig to non-human-primate renal xenotransplantation: Systematic review and comparative outcomes analysis of 1051 life-sustaining NHP renal allo- and xeno-transplants. Am J Transpl. (2022) 22:1527–36. doi: 10.1111/ajt.16994, PMID: 35143091

[45] Cooper DKC Riella LV Kawai T Fishman JA Williams WW Elias N. The time has come: the case for initiating pilot clinical trials of pig kidney xenotransplantation. Ann Surg. (2024) 281:204–9. doi: 10.1097/SLA.0000000000006529, PMID: 39263749 PMC11723495

[46] Heo Y Cho Y Oh KB Park KH Hansam C Choi H. Detection of pig cells harboring porcine endogenous retroviruses in non-human primate bladder after renal xenotransplantation. Viruses. (2019) 11:801. doi: 10.3390/v11090801, PMID: 31470671 PMC6784250

[47] Yang C Wei Y Li X Xu K Huo X Chen G. Production of four-gene (GTKO/hCD55/hTBM/hCD39)-edited donor pigs and kidney xenotransplantation. Xenotransplantation. (2024) 31:e12881. doi: 10.1111/xen.12881, PMID: 39185796

[48] Wang Y Chen G Pan D Guo H Jiang H Wang J. Pig-to-human kidney xenotransplants using genetically modified minipigs. Cell Rep Med. (2024) 5:101744. doi: 10.1016/j.xcrm.2024, PMID: 39317190 PMC11513830

[49] Eisenson D Hisadome Y Santillan M Iwase H Chen W Shimizu A. Consistent survival in consecutive cases of life-supporting porcine kidney xenotransplantation using 10GE source pigs. Nat Commun. (2024) 15:3361. doi: 10.1038/s41467-024-47679-6, PMID: 38637524 PMC11026402

[50] Judd E Kumar V Porrett PM Hyndman KA Anderson DJ Jones-Carr ME. Physiologic homeostasis after pig-to-human kidney xenotransplantation. Kidney Int. (2024) 105:971–9. doi: 10.1016/j.kint.2024.01.016, PMID: 38290599 PMC11457287

[51] Längin M Bender M Schmoeckel M Reichart B. Progress in orthotopic pig heart transplantation in nonhuman primates. Transpl Int. (2024) 37:13607. doi: 10.3389/ti.2024.13607, PMID: 39399753 PMC11466817

[52] Kuwaki K Tseng YL Dor FJMF Shimizu A Houser SL Sanderson TM. Heart transplantation in baboons using alpha1,3-galactosyltransferase gene-knockout pigs as donors: initial experience. Nat Med. (2005) 11:29–31. doi: 10.1038/nm1171, PMID: 15619628

[53] Lila N McGregor CGA Carpentier S Rancic J Byrne GW Carpentier A. Gal knockout pig pericardium: new source of material for heart valve bioprostheses. J Heart Lung Transpl. (2010) 29:538–43. doi: 10.1016/j.healun.2009.10.007, PMID: 20036160

[54] Diswall M Angström J Karlsson H Phelps CJ Ayares D Teneberg S. Structural characterization of alpha1,3-galactosyltransferase knockout pig heart and kidney glycolipids and their reactivity with human and baboon antibodies. Xenotransplantation. (2010) 17:48–60. doi: 10.1111/j.1399-3089.2009.00564.x, PMID: 20149188

[55] Mohiuddin MM Corcoran PC Singh AK Azimzadeh A Hoyt RF Jr. Thomas ML. B-cell depletion extends the survival of GTKO.hCD46Tg pig heart xenografts in baboons for up to 8 months. Am J Transpl. (2012) 12:763–71. doi: 10.1111/j.1600-6143.2011.03846.x, PMID: 22070772 PMC4182960

[56] Azimzadeh AA Kelishadi SS Ezzelarab MB Singh AK Stoddard T Iwase H. Early graft failure of GalTKO pig organs in baboons is reduced by expression of a human complement pathway-regulatory protein. Xenotransplantation. (2015) 22:310–6. doi: 10.1111/xen.12176, PMID: 26174749 PMC5172381

[57] Mcgregor C Byrne G Rahmani B Chisari E Kyriakopoulou K Burriesci G. Physical equivalency of wild type and galactose α 1,3 galactose free porcine pericardium; a new source material for bioprosthetic heart valves. Acta Biomater. (2016) 41:204–9. doi: 10.1016/j.actbio.2016.06.007, PMID: 27268480 PMC4982525

[58] Abicht JM Sfriso R Reichart B Längin M Gahle K Puga Yung GL. Multiple genetically modified GTKO/hCD46/HLA-E/hβ2-mg porcine hearts are protected from complement activation and natural killer cell infiltration during ex vivo perfusion with human blood. Xenotransplantation. (2018) 25:e12390. doi: 10.1111/xen.12390, PMID: 29536572

[59] Griffith BP Goerlish CE Singh AK Rothblatt M Lau CL Shah A. Genetically modified porcine-to-human cardiac xenotransplantation. N Engl J Med. (2022) 387:35–44. doi: 10.1056/NEJMoa2201422, PMID: 35731912 PMC10361070

[60] Mohiuddin MM Goerlich CE Singh AK Zhang T Tatarov I Lewis B. Progressive genetic modifications of porcine cardiac xenografts extend survival to 9 months. Xenotransplantation. (2022) 29:e12744. doi: 10.1111/xen.12744, PMID: 35357044 PMC10325874

[61] Mohiuddin MM Singh AK Scobie L Goerlich CE Grazioli A Saharia K. Graft dysfunction in compassionate use of genetically engineered pig-to-human cardiac xenotransplantation: a case report. Lancet. (2023) 402:397–410. doi: 10.1016/S0140-6736(23)00775-4, PMID: 37393920 PMC10552929

[62] Moazami N Stern JM Khalil K Kim JI Narula N Mangiola M. Pig-to-human heart xenotransplantation in two recently deceased human recipients. Nat Med. (2023) 29:1989–97. doi: 10.1038/s41591-023-02471-9, PMID: 37488288

[63] Singh AK Goerlich CE Zhang T Lewis B Hershfeld A Braileanu G. Genetically engineered pig heart transplantation in non-human primates. Commun Med (Lond). (2025) 5:6. doi: 10.1038/s43856-025-00731-y, PMID: 39774817 PMC11707197

[64] Griffith BP Grazioli A Singh AK Tully A Galindo J Saharia KK. Transplantation of a genetically modified porcine heart into a live human. Nat Med. (2025) 31:589–98. doi: 10.1038/s41591-024-03429-1, PMID: 39779924

[65] Gao M Zhang J Wang R Yang G Bao J. Pig-to-Human xenotransplantation: Moving toward organ customization. Precis Clin Med. (2023) 6:pbad013. doi: 10.1093/pcmedi/pbad013, PMID: 37324749 PMC10266333

[66] Yuan Y Cui Y Zhao D Yuan Y Zhao Y Li D. Complement networks in gene-edited pig xenotransplantation: enhancing transplant success and addressing organ shortage. J Transl Med. (2024) 22:324. doi: 10.1186/s12967-024-05136-4, PMID: 38566098 PMC10986007

[67] Chaban R Ileka IS Pierson RN III. Lung xenotransplantation: current status 2023. Eur J Transplant. (2023), 217–25. doi: 10.57603/EJT-311

[68] Westall GP Levvey BJ Salvaris E Gooi J Marasco S Rosenfeldt F. Sustained function of genetically modified porcine lungs in an ex vivo model of pulmonary xenotransplantation. J Heart Lung Transpl. (2013) 32:1123–30. doi: 10.1016/j.healun.2013.07.001, PMID: 23932853

[69] Platz J Bonenfant NR Uhl FE Coffey AL McKnight T Parsons C. Comparative decellularization and recellularization of wild-type and alpha 1,3 galactosyltransferase knockout pig lungs: A model for ex vivo xenogeneic lung bioengineering and transplantation. Tissue Eng Part C Methods. (2016) 22:725–39. doi: 10.1089/ten.TEC.2016.0109, PMID: 27310581 PMC4991572

[70] Stahl EC Bonvillain RW Skillen CD Burger BL Hara H Lee W. Evaluation of the host immune response to decellularized lung scaffolds derived from α-Gal knockout pigs in a non-human primate model. Biomaterials. (2018) 187:93–104. doi: 10.1016/j.biomaterials.2018.09.038, PMID: 30312852

[71] Watanabe H Ariyoshi Y Pomposelli T Takeuchi K Ekanayake-Alper DK Boyd LK. Intra-bone bone marrow transplantation from hCD47 transgenic pigs to baboons prolongs chimerism to >60 days and promotes increased porcine lung transplant survival. Xenotransplantation. (2020) 27:e12552. doi: 10.1111/xen.12552, PMID: 31544995 PMC7007336

[72] Gasek N Dearborn J Enes SR Pouliot R Louie J Phillips Z. Comparative immunogenicity of decellularized wild type and alpha 1,3 galactosyltransferase knockout pig lungs. Biomaterials. (2021) 276:121029. doi: 10.1016/j.biomaterials.2021.121029, PMID: 34311317

[73] Burdorf L Laird CT Harris DG Connolly MR Habibabady Redding E. Pig-to-baboon lung xenotransplantation: Extended survival with targeted genetic modifications and pharmacologic treatments. Am J Transpl. (2022) 22:28–45. doi: 10.1111/ajt.16809, PMID: 34424601 PMC10292947

[74] Chaban R McGrath G Habibabady Z Rosales I Burdorf L Ayares DL. Increased human complement pathway regulatory protein gene dose is associated with increased endothelial expression and prolonged survival during ex-vivo perfusion of GTKO pig lungs with human blood. Xenotransplantation. (2023) 30:e12812. doi: 10.1111/xen.12812, PMID: 37504492 PMC12882787

[75] Choi HJ Kim MK Lee HJ Jeong SH Kang HJ Park CS. Effect of αGal on corneal xenotransplantation in a mouse model. Xenotransplantation. (2011) 18:176–82. doi: 10.1111/j.1399-3089.2011.00641.x, PMID: 21696447

[76] Hara H Koike N Long C Piluek J Roh SD SundarRaj N. Initial *in vitro* investigation of the human immune response to corneal cells from genetically engineered pigs. Invest Ophthalmol Vis Sci. (2011) 52:5278–86. doi: 10.1167/iovs.10-6947, PMID: 21596821 PMC3176056

[77] Lee W Miyagawa Y Long C Cooper DKC Hara H. A comparison of three methods of decellularization of pig corneas to reduce immunogenicity. Int J Ophthalmol. (2014) 7:587–93. doi: 10.3980/j.issn.2222-3959.2014.04.01, PMID: 25161926 PMC4137190

[78] Lee W Miyagawa Y Long C Ekser B Walters E Ramsoondar J. Expression of neuGc on pig corneas and its potential significance in pig corneal xenotransplantation. Cornea. (2016) 35:105–13. doi: 10.1097/ICO.0000000000000635, PMID: 26418433 PMC4847538

[79] Dong X Hara H Wang Y Wang L Zhang Y Cooper DKC. Initial study of α1,3-galactosyltransferase gene-knockout/CD46 pig full-thickness corneal xenografts in rhesus monkeys. Xenotransplantation. (2017) 24. doi: 10.1111/xen.12282, PMID: 28054735

[80] Yoon CH Choi SH Choi HJ Hyun JL Hee JK Jong MK. Long-term survival of full-thickness corneal xenografts from α1,3-galactosyltransferase gene-knockout miniature pigs in non-human primates. Xenotransplantation. (2020) 27:e12559. doi: 10.1111/xen.12559, PMID: 31566261

[81] Kim JW Kim HM Lee SM Kang MJ. Porcine knock-in fibroblasts expressing hDAF on α-1,3-galactosyltransferase (GGTA1) gene locus. Asian-Australas J Anim Sci. (2012) 25:1473–80. doi: 10.5713/ajas.2012.12146, PMID: 25049505 PMC4093019

[82] Liu Y Yang JY Lu Y Yu P Dove R Hutcheson JM. α-1,3-Galactosyltransferase knockout pig induced pluripotent stem cells: a cell source for the production of xenotransplant pigs. Cell Reprogram. (2013) 15:107–16. doi: 10.1089/cell.2012.0062, PMID: 23402576

[83] Kumar G Hara H Long C Shaikh H Ayares D Cooper DKC. Adipose-derived mesenchymal stromal cells from genetically modified pigs: immunogenicity and immune modulatory properties. Cytotherapy. (2012) 14:494–504. doi: 10.3109/14653249.2011.651529, PMID: 22264190 PMC3774176

[84] Schubert T Poilvache H Galli C Gianello P Dufrane D. Galactosyl-knock-out engineered pig as a xenogenic donor source of adipose MSCs for bone regeneration. Biomaterials. (2013) 34:3279–89. doi: 10.1016/j.biomaterials.2013.01.057, PMID: 23375391

[85] Lee W Hara H Ezzelarab MB Iwase H Bottino R Long C. Initial *in vitro* studies on tissues and cells from GTKO/CD46/NeuGcKO pigs. Xenotransplantation. (2016) 23:137–50. doi: 10.1111/xen.12229, PMID: 26988899 PMC4842123

[86] Bongoni AK Salvaris E Nordling S Klymiuk N Wolf E Ayares DL. Surface modification of pig endothelial cells with a branched heparin conjugate improves their compatibility with human blood. Sci Rep. (2017) 7:4450. doi: 10.1038/s41598-017-04898-w, PMID: 28667310 PMC5493627

[87] Hara H Iwase H Nguyen H Miyagawa Y Kuravi K Foote JB. table expression of the human thrombomodulin transgene in pig endothelial cells is associated with a reduction in the inflammatory response. Cytokine. (2021) 148:155580. doi: 10.1016/j.cyto.2021.155580, PMID: 34099346 PMC8511266

[88] Huai G Wang Y Du J Cheng Z Xie Y Zhou J. The generation and evaluation of TKO/hCD55/hTM/hEPCR gene-modified pigs for clinical organ xenotransplantation. Front Immunol. (2025) 15:1488552. doi: 10.3389/fimmu.2024.1488552, PMID: 39902050 PMC11788277

[89] Eisenson D Hisadome Y Yamada L. Progress in xenotransplantation: immunologic barriers, advances in gene editing, and successful tolerance induction strategies in pig-to-primate transplantation. Front Immunol. (2022) 13:899657. doi: 10.3389/fimmu.2022.899657, PMID: 35663933 PMC9157571

[90] Kuravi K Sorrells L Nellis JR Rahman F Walters AH Matheny RG. Allergic response to medical products in patients with alpha-gal syndrome. J Thorac Cardiovasc Surg. (2022) 164:e411–24. doi: 10.1016/j.jtcvs.2021.03.100, PMID: 33933257 PMC9673037

[91] Cooper DKC Dorling A Pierson RN 3rd Rees M Seebach J Yazer M. Alpha1,3-galactosyltransferase gene-knockout pigs for xenotransplantation: where do we go from here? Transplantation. (2007) 84:1–7. doi: 10.1097/01.tp.0000260427.75804.f2, PMID: 17627227

[92] Zeyland J Wozniak A Gawronska B Juzwa W Jura J Nowak A. Double transgenic pigs with combined expression of human α1,2-fucosyltransferase and α-galactosidase designed to avoid hyperacute xenograft rejection. Arch Immunol Ther Exp (Warsz). (2014) 62:411–22. doi: 10.1007/s00005-014-0280-3, PMID: 24554032 PMC4164832

[93] Cooper DKC Hara H. You cannot stay in the laboratory forever”*: Taking pig kidney xenotransplantation from the laboratory to the clinic. EBioMedicine. (2021) 71:103562. doi: 10.1016/j.ebiom.2021.103562, PMID: 34517284 PMC8441149

[94] Zhang J Xie C Lu Y Zhou M Qu Z Yao D. Potential antigens involved in delayed xenograft rejection in a Ggta1/Cmah Dko pig-to-monkey model. Sci Rep. (2017) 7:10024. doi: 10.1038/s41598-017-10805-0, PMID: 28855711 PMC5577312

[95] Cooper DK Wang L Kinoshita K Habibabady Z Rosales I Kobayashi T. Immunobiological barriers to pig organ xenotransplantation. Eur J Transplant. (2023), 167–81. doi: 10.57603/EJT-266

[96] Wu H Lian M Lai L. Multiple gene modifications of pigs for overcoming obstacles of Xenotransplantation. Natl Sci Open. (2023) 2:20230030. doi: 10.1360/nso/20230030

[97] Ekser B Rigotti P Gridelli B Cooper DKC. Xenotransplantation of solid organs in the pig-to-primate model. Transpl Immunol. (2009) 21:87–92. doi: 10.1016/j.trim.2008.10.005, PMID: 18955143

